# Serum Retinol Binding Protein as an Indicator of Vitamin A Status in Cirrhotic Patients with Night Blindness

**DOI:** 10.4103/1319-3767.37794

**Published:** 2008-01

**Authors:** Khalid Mahmood, Akhtar H. Samo, Krishan L. Jairamani, Gohar Ali, Abu Talib, Waqar Qazmi

**Affiliations:** Medical Unit IV, Dow University of Health Sciences, Karachi, Pakistan

**Keywords:** Cirrhosis, hypovitaminosis A, night blindness, retinol binding protein

## Abstract

**Background/Aim:**

Vitamin A deficiency is known to be associated with night blindness. Plasma retinol binding protein (RBP) estimation highly correlates with plasma retinol concentration to predict vitamin A status. Serum RBP estimation is reasonably simple, inexpensive, and highly applicable in less technologically developed settings. We studied the correlation of plasma vitamin A levels (by RBP estimation) and ocular manifestation in patients with liver cirrhosis.

**Materials and Methods:**

This prospective, cohort study included 137 patients with liver cirrhosis. Ocular manifestations in these patients were recorded along with detailed history and clinical examination. Blood samples after overnight fasting were measured for RBP levels. The characteristics of cirrhotic patients with and without eye findings were compared.

**Results:**

Out of 137 patients, 55% were males. The causes of cirrhosis were hepatitis C virus in 61%, hepatitis B virus in 32%, alcoholics in 3%, and primary biliary cirrhosis in 3%. Ocular manifestations were found in 47% patients. RBP levels were found to be low in 44%, normal in 40%, and relatively high in 16% patients. Low levels of RBP compared to normal were associated with opthalmological findings of hypovitaminosis A (*P* < 0.001).

**Conclusion:**

The measurement of plasma RBP as an alternative to serum retinol estimation to detect relative hypovitaminosis A is simple, easy and reliable. Vitamin A level is strongly related to the severity of liver disease. Opthalmological manifestations in patients with liver cirrhosis may be preventable by early detection of hypovitaminosis A with serum RBP level, but larger studies are required before recommendation of vitamin A supplementation.

Vitamin A is an essential part of rhodopsin, a retinal pigment required for dim light vision (dark adaptation). Vitamin A exists in the diet as retinyl esters or carotenoids (provitamin A); these are metabolized to retinol in the intestinal mucosa, where it is esterified and transported through chylomicrons into the circulation. Most of the esters are taken up and stored by the liver cells; 90% of vitamin A is being stored in liver.[1] Vitamin A is transported to the tissues in the form of retinol bound to retinol-binding proteins (RBP) in a 1:1 complex. Normally, 95% of vitamin A circulating in the plasma comprises of retinol bound to RBP, and the remaining 5% constitutes retinyl esters in chylomicrons. Plasma vitamin A levels are largely regulated by the production and turnover rates of RBP. Plasma RBP concentrations measured by radial immunodiffusion highly correlate with plasma retinol[2] as both exist in the circulation in equimolar concentrations. Plasma RBP has 93% sensitivity for predicting marginal vitamin A status and 91% sensitivity for predicting vitamin A deficiency; the corresponding specificities are 75% and 94%, respectively.[3] RBP is less photo- and heat-sensitive than retinol,[4] where laboratory and refrigeration facilities are poor. More accurate results may be obtained by measuring RBP rather than retinol concentrations.

Vitamin A deficiency leads to night blindness and xerophthalmia in the form of Bitot's spots, xerosis, keratomalacia, and even retinopathy.[5,6] Vitamin A deficiency is common in children in the poor, underdeveloped and developing countries.

Liver injury secondary to chronic hepatitis B virus (HBV)[7] and hepatitis C virus (HCV)[8] infections is prevalent worldwide and is a leading cause of liver cirrhosis and hepatocellular carcinoma.[9,10] Xerophthalmia and color blindness due to vitamin A deficiency are not rare findings in cirrhosis of liver. Cirrhosis of liver in itself is well known to cause malnutrition and malabsorption, especially of fat-soluble vitamins and also reduces the capacity of liver to store these vitamins.[11] Decreased storage of vitamin A in liver and its malabsorption are responsible for deficiency of vitamin A in cirrhotics. Color blindness is more common in alcoholic type of cirrhosis.[12] Night blindness is a common finding in cirrhosis of liver and its relationship to plasma vitamin A levels has not been studied extensively.

Serum RBP is a sensitive and specific surrogate marker for serum retinol, which is reasonably simple, inexpensive and highly applicable in less technologically advanced settings. Hence, we conducted this study to investigate the correlation of plasma vitamin A levels by RBP estimation with night blindness in patients with liver cirrhosis.

## MATERIALS AND METHODS

### Patient population

This prospective, cohort study was conducted at a tertiary care teaching hospital in Karachi, the Civil Hospital attached to the Dow University of Health Sciences. All eligible patients admitted to the hospital from 6 July, 2004 to 31 May, 2006 with a diagnosis of cirrhosis of liver were enrolled in the study. Patients having diabetes mellitus, hypertension, ischemic and non-ischemic retinopathy, retinitis pigmentosa and those on vitamin A supplementation during the last 2 months were excluded from the study. Patients on drugs, which could interfere with vitamin A metabolism like neomycin, cholestyramine, etc., were also excluded from the study.

### Definitions

#### Cirrhosis of liver

The diagnosis of cirrhosis of liver was made on the basis of biopsy, clinical findings, and ultrasonic features. Ten patients were proven by liver biopsy while others had clinical (portal hypertension, ascites, varices with peripheral signs of chronic liver disease), laboratory [low serum albumin, abnormal prothrombin time (PT) and liver function tests (LFTs)], and ultrasonographic features (small liver with altered echogenicity, dilated portal vein > 1.4 cm, splenomegaly, ascites, splenic varices) compatible with cirrhosis of liver.

#### Child-Pugh classification

Patients were also classified according to the Child-Pugh classification. The Child-Pugh classification has been designed to assess the prognosis of patients with cirrhosis of liver. This classification is based on scoring system that incorporates ascites, hepatic encephalopathy, serum albumin, serum bilirubin and prothrombin time, which are well-known indicators of prognosis. Score between 1 and 3 is awarded to each variable according to severity. The score from 5 to 6 makes class A with relatively fair prognosis, 7–9 is class B, and 10–15 is class C, having worse prognosis.

#### WHO classification of ocular manifestations in hypovitaminosis A

The ocular manifestations in patients with hypovitaminosis A have been classified by WHO as follows:

H/O Night Blindness (XN)
Conjunctival Xerosis (XIA)
Conjunctival Xerosis with Bitot's Spots (XIB)
Corneal Xerosis (X2)
Corneal Scars (XS)

#### Clinical findings and laboratory investigations

A detailed history was taken and thorough clinical examination was performed. Relevant laboratory investigations including complete blood counts (CBC), LFTs, viral markers (HCV and HBV), serum proteins with A:G ratio, PT, blood sugar (RBS), urea, creatinine, serum electrolytes and sonography of abdomen was performed in every patient. A fundoscopic and slit lamp examination was performed following pupil dilatation with 1% tropicamide. Overnight fasting blood samples were collected for RBP and quantitatively measured by ELISA technique using DRG diagnostics instrument (Gmbh, Germany). The normal reference value of RBP used was 30–75 mg/l.

#### Informed consent

An informed written consent from every patient and approval of Ethical Committee of Dow University was taken.

### Statistical analysis

#### Descriptive analyses

A descriptive analysis of continuous and categorical variables was performed. Continuous variables were expressed as means ± SD and categorical variables were expressed as proportions (%). Patient characteristics including age, gender, etiology of cirrhosis, levels of RBP, and eye findings were determined.

Comparisons were made between the characteristics of cirrhotic patients with eye findings and those without eye findings. Chi-square tests were performed for categorical variables and *t*-tests for continuous variables. *P* < 0.05 was considered to be statistically significant. Statistical analyses were performed using SPSS Version 13.0.

## RESULTS

**Figure 1:**
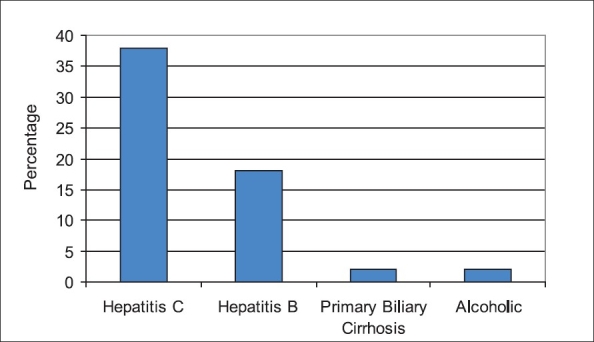
Etiology of cirrhosis of liver in patients with low RBP levels

A total of 137 patients with a diagnosis of cirrhosis of the liver were included in the study. Out of 137 patients, 55% were males. The mean age for males was 56 ± 9 years and those of females was 54 ± 8 years. The etiological breakdown for cirrhosis of liver was as follows: HCV 84 (61%), HBV 44 (32%), alcoholics 5 (4%) and primary biliary cirrhosis (PBC) 4 (3%) [Figure 1]. According to Child-Pugh classification, 15 patients were in class A, 39 in class B, and 83 in class C [Table 1].

**Table 1:** Demographic, clinical and laboratory features of patients with cirrhosis of liver

Patient characteristics	(*n*)% or mean ± SD
Age (years)	54.9 ± 8.5
Gender (male %)	55%
Etiology	
HCV	84 (61)
HBV	44 (32)
Primary biliary cirrhosis	4 (3)
Alcoholic	5 (4)
Child-Pugh class	
C	83 (61)
B	39 (28)
A	15 (11)
RBP levels	
Low	60 (44)
Normal	55 (40)
High	22 (16)
Eye findings	
Positive	64 (47)
Negative	73 (53)

Figures in parentheses are in percentage

### Eye findings

History of night blindness was present in 64 (47%) patients; of these, nine had conjunctival xerosis, five had conjunctival xerosis with Bitot's spots, but none of these patients had corneal xerosis or corneal scar, according to the WHO classification.

### RBP level and eye findings

RBP level was found to be low in 60 (44%) patients, normal in 55 (40%) patients, and relatively high in 22 (16 %) patients. All the patients with low levels of RBP were those having opthalmological findings of hypovitaminosis A, and four patients with night blindness had normal RBP level, proving a strong correlation between low RBP and opthalmological findings (*P* < 0.001).

### Etiology of cirrhosis and low RBP level

**Figure 2:**
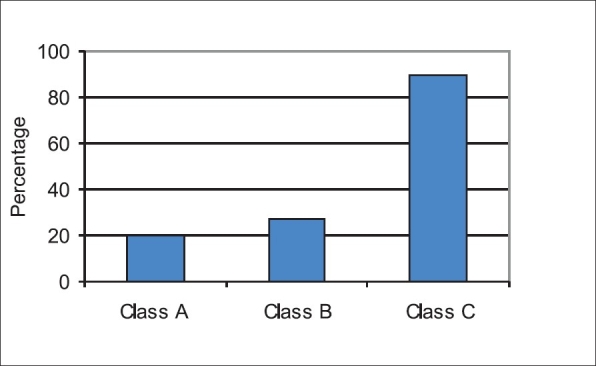
Child-Pugh classification of patients with low RBP levels

The etiology of patients with cirrhosis having low RBP [Figure 2] included HCV in 39 patients, HBV in 19 patients, PBC in 1 patient and alcohol in 1 patient.

### Child-Pugh classification and RBP level

**Figure 3:**
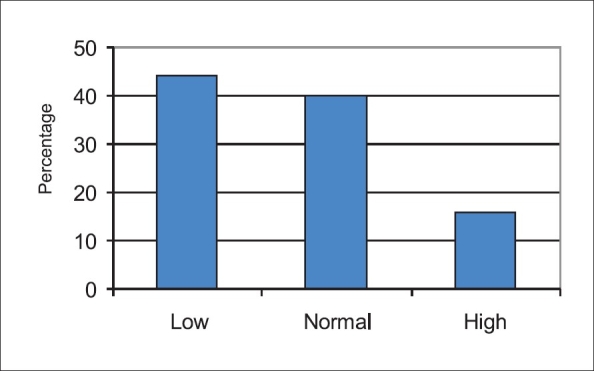
RBP levels in patients with cirrhosis of liver

Child-Pugh classification of these patients revealed that 42 out of 60 patients with low RBP were in class C, 16 were in class B, and 2 were in class A [Figure 3], showing a strong correlation between low levels of RBP and Child-Pugh class C [Tables 2 and 3].

**Table 2:** Etiology of cirrhosis and Child-Pugh classification by serum RBP levels in patients with cirrhosis of liver

	RBP levels low (*n* = 60)	RBP levels normal (*n* = 55)	RBP levels high (*n* = 22)
Etiology of cirrhosis			
HCV	39 (46)	35 (42)	10 (12)
HBV	19 (43)	16 (36)	9 (20)
Biliary cirrhosis	1 (25)	3 (75)	0
Alcoholic	1 (20)	1 (20)	3 (60)
Child-Pugh class			
C	42 (51)	31 (37)	10 (2)
B	16 (4)	16 (4)	7 (18)
A	2 (13)	8 (53)	5 (33)

Figures in parentheses are in percentage

**Table 3:** Comparison of patients with and without eye findings by etiology of cirrhosis, Child-Pugh class and RBP levels

	Eye findings positive (*n* = 64)	Eye findings negative (*n* = 73)	*P*-value
Etiology of cirrhosis			
HCV	41 (48)	43 (52)	NS
HBV	19 (43)	25 (57)	NS
Primary biliary cirrhosis	3 (75)	1 (25)	NS
Alcoholic	1 (20)	4 (80)	NS
Child-Pugh class			
C	44 (53)	39 (47)	NS
B	17 (43)	22 (57)	NS
A	3 (20)	12 (80)	0.006
RBP levels			
Low	60 (100)	0	<0.001
Normal	4 (7)	51 (93)	<0.001
High	0	22 (100)	<0.001

Figures in parentheses are in percentage

## DISCUSSION

The topic studied is unique in a sense that most of the previous studies, which have been conducted in India, Pakistan, Bangladesh, Indonesia, and Philippines,[13–18] have featured children and hypovitaminosis A was found secondary to nutritional deficiencies. There is scarce material available on this topic wherein only a few studies were conducted over two decades ago.

This study revealed that males comprised 55% of the total cirrhotic patients included in this study, reflecting overall prevalence of chronic HBV and HCV infections in males. Most of the studies on prevalence of cirrhosis of liver have similarly shown males to be the preponderant gender.[12]

Serum RBP levels were low in 44% patients proving the fact that RBP and hence vitamin A deficiency is common in patients with cirrhosis of liver. A previous study by Zuberi *et al*. had reported low vitamin A levels in 75% of the cirrhotics;[19] two other studies, one reported in 1939 by Patek and Haig and the other in 1948 by Adlersberg also showed similar results pertaining to deficiency of vitamin A in cirrhotics.[20,21] In another study, Ukleja ***et al.*** had shown significantly low serum retinol concentration in cirrhotic patients.[22]

In this study, the serum RBP levels were normal in 40% patients and remaining 16% patients had rather high levels of RBP. A similar finding has also been reported in a previous study by Ukleja *et al.*[22] in 2002 and this high fasting concentration of vitamin A may reflect reduced storage capacity of damaged liver, delayed postprandial chylomicron clearance, reduced hepatic uptake of vitamin A as a result of ongoing liver damage and inadvertent vitamin A supplementation, although this was an exclusion criteria in this study.

In the present study, 64 patients had night blindness. However, RBP level was low in 60 patients, the remaining 4 patients with night blindness had normal RBP level, two of the patients were suffering from PBC with Child-Pugh classes A and B, respectively, and other two patients had HCV as a cause of cirrhosis with Child-Pugh class C. Similar results have been reported by Ukleja *et al.*[22] with normal vitamin A levels and night blindness in some cirrhotic patients, who instead had low hepatic vitamin A levels, indicating the complexity of vitamin A metabolism in cirrhotic patients.

This study also revealed that all of the patients with low serum RBP levels had eye findings of vitamin A deficiency in the form of night blindness, conjunctival xerosis in 9/60 patients, and conjunctival xerosis with Bitot's spots in 5/60 patients. These results are statistically significant and are consistent with the study conducted by Patek and Haig[20] who also found high prevalence of night blindness in patients with cirrhosis of liver, which effectively reversed on vitamin A supplementation. On the contrary, another study conducted by Ukleja *et al.*,[22] although found low serum concentration of retinol in cirrhotic patients, did not find a significant difference in serum vitamin A concentration in those having symptoms of night blindness and those without. However, total hepatic vitamin A levels were significantly lower in patients with symptoms of night blindness than in those without, again proving the association between vitamin A deficiency and night blindness.

In this study, the Child-Pugh classification of the patients with low serum RBP levels revealed that 70% belonged to class C, 27% to class B, and only 3% to class A, indicating a strong association between low RBP level and severity of liver disease. In the present study, Child-Pugh classification and low levels of RBP were found to be significantly associated with eye findings in patients with cirrhosis of the liver. No comparative data are available on this aspect of study in recent literature.

Chronic hepatitis C infection was found to be the most prevalent cause of liver cirrhosis, followed by chronic HBV, alcohol, and PBC reflecting the overall prevalence of these etiological causes for cirrhosis of liver in this part of world.[23] Serum RBP level was found to be low, more commonly in patients with cirrhosis caused by HCV (65%) and HBV (32%) infections. Ukleja *et al.* have also shown low levels of vitamin A to be more prevalent in cirrhosis due to HCV in their study.[22]

Regarding the prevalence of ocular manifestations of vitamin A deficiency, this study determined that night blindness (XN) was present in all 60/60 (100%) patients. In patients having hypovitaminosis A, indirectly measured by serum RBP level, 9 out of 60 patients had conjunctival xerosis (X1A) and 5 had conjunctival xerosis with Bitot's spots (X1B), but none had corneal xerosis (X2) or corneal scars (XS), in accordance with the WHO classification. Most of the previous studies have targeted dark adaptation time/night blindness against serum/hepatic vitamin A level in cirrhotic patients.[24] However, a study by Ngah *et al.*, conducted on children for ocular manifestations of vitamin A deficiency, revealed that night blindness was present in overall 16% of the children, conjunctival xerosis in 57%, Bitot's spots in 3%, corneal xerosis in 0.5%, and corneal scar in 6%; and overall 82% of the children had eye manifestation of vitamin A deficiency.[25] This study shows that night blindness was significantly associated with low RBP levels. There was a 100% sensitivity of low serum RBP levels and the diagnosis of ocular manifestations; however, four patients with normal RBP levels had ocular manifestations. These findings are similar to an earlier study by Ngah and colleagues.[25]

## CONCLUSION

Measurement of plasma retinol level requires complicated and expensive laboratory equipments and specific reagents. Measurement of RBP is relatively simple, cheap with high specificity and sensitivity, and can be applied in less advanced laboratory set-ups with equally good results. Vitamin A deficiency is prevalent in patients with liver cirrhosis, being more common in patients with Child-Pugh classes C and B, and night blindness is an early manifestation carrying high predictive value for low serum RBP levels, which is an indirect indicator of vitamin A deficiency. But it is premature to recommend vitamin A supplementation, further extensive studies based on comparative RBP and serum retinol measurement along with hepatic vitamin A level and their correlation with opthalmological findings are required.

## References

[1] Goodman DS (1984). Vitamin A and retinoids in health and disease. N Engl J Med.

[2] Almekinder J, Manda W, Soko D, Lan Y, Hoover DR, Semba RD (2000). Evaluation of plasma retinol-binding protein as a surrogate measure for plasma retinol concentrations. Scand J Clin Lab Invest.

[3] Baeten JM, Richardson BA, Bankson DD, Wener MH, Kreiss JK, Lavreys L (2004). Use of serum retinol-binding protein for prediction of vitamin A deficiency: Effects of HIV-1 infection, protein malnutrition and the acute phase response. Am J Clin Nutr.

[4] Gamble MV, Ramakrishnan R, Palafox NA, Briand K, Berglund L, Blaner WS (2001). Retinol binding protein as a surrogate measure for serum retinol: Studies in vitamin A-deficient children from the Republic of the Marshall Islands. Am J Clin Nutr.

[5] Etti A, Daxecker F (1992). Xeropthalmia in liver cirrhosis: Correct diagnosis after 15 years. Opthlmologica.

[6] McClain CJ, Van Thiel DH, Parker S, Badzin LK, Gilbert H (1979). Alteration in Zinc, vitamin A and retinol binding protein in chronic alcoholics: A possible mechanism for night blindness and hypogonadism. Alcohol Clin Exp Res.

[7] Abbas Z, Jafri, Shah SH, Khokhar N, Zuberi SJ (2004). Pakistan Society of Gestroenterology and GI Endoscopy: PGS Consenses Statement on management of hepatitis B Virus infection, 2003. J Pak Med Assoc.

[8] Hamid S, Umer M, Aslam A, Siddique A, Qureshi H, Butt J (2004). PGS consenses statement on management of hepatitis C virus infection-2003. J Pak Med Assoc.

[9] Seeff LB (1997). Natural history of hepatitis C. Hepatology.

[10] Lee WM (1997). Hepatitis B virus infection. N Engl J Med.

[11] Underwood BA, Sporn MB, Roberts AB, Goodman DS (1984). Vitamin A in animal and human nutrition. The Retinoids.

[12] Onder C, Bengur T, Selcuk D, Balents, Bellcis U, Ahmet M (2005). Relationship between retinopathy and cirrhosis. World J Gastroenterol.

[13] Gujral S, Abbi R, Gopaldas T (1993). Xeropthalmia, vitamin A supplementation and morbidity in children. J Trop Paediatr.

[14] Molla A, Badruddin SH, Khurshid M, Molla AM, Rehman FN, Durrani S (1993). Vitamin A status of children in the urban slums of Karachi, Pakistan, assessed by clinical, dietry and biochemical methods. Am J Trop Med Hyg.

[15] Copen N, Measham C, Khanusar S, Khatum M, Ahmed N (1983). Xeropthalmia in urban Bangladesh: Implication for vitamin A deficiency strategies. Acta Paedia Scand.

[16] Tarwotjo I, Katz J, West KP (1992). Xeropthalmia and growth in preschool Indonesian children. Am J Clinc Nutr.

[17] Gilbert C, Foster A (1993). Causes of blindness attending four school for the blind in Thialand and Philippines. Int Opthal.

[18] Khandait DW, Vasudeo ND, Zodpey SP, Ambadekr NN, Koram MR (1999). Vitamin A intake and xerophthalmia among Indian children. Public health.

[19] Zuberi SJ, Ibrahim K (2004). Palsma Vit A level in hepatitis and cirrhosis. J Pak Med Assoc.

[20] Patek AJ, Haig C (1939). The occurrence of abnormal dark adaptation and its relation to vitamin A metabolism in patients with cirrhosis of the liver. J Clin Invest.

[21] Adlersberg D, Kann S, Maurer AP, Newerly K, Winternitz W, Sobotka H (1948). Vitamin A metabolism in liver disease. Gastroenterology.

[22] Ukleja A, Scolapio JS, McConnell JP, Spivey JR, Dickson RC, Nguyen JH (2002). Nutrition assessment of serum and hepatic vitamin A levels in patients with cirrhosis. JPEN J Parenter Enteral Nutr.

[23] Vaiphei K, Pal NS, Arora SK (2006). Comparative analysis of HBV and HCV infection in hepatocellular carcinoma and chronic liver disease: An autopsy based study. Indian J Pathol Microbiol.

[24] Russell RM, Morrison SA, Smith FR, Oaks EV, Carney EA (1978). Vitamin A reversal of abnormal dark adaptation in cirrhosis: Study of effects on the plasma retinol transport system. Ann Intern Med.

[25] Ngah NF, Moktar N, Isa NH, Selvara S, Yusof MS, Sani HA (2002). Ocular manifestation of vitamin A deficiency among Orang Asli (Aborigine) children in Malaysia. Asia Pac J Clin Nutr.

